# Theory of the deformation of aligned polyethylene

**DOI:** 10.1098/rspa.2015.0171

**Published:** 2015-08-08

**Authors:** A. Hammad, T. D. Swinburne, H. Hasan, S. Del Rosso, L. Iannucci, A. P. Sutton

**Affiliations:** 1 Department of Physics, Imperial College London, Exhibition Road, London SW7 2AZ, UK; 2 Department of Aeronautics, Imperial College London, Exhibition Road, London SW7 2AZ, UK

**Keywords:** aligned polyethylene, plastic deformation, viscoelasticity, Frenkel–Kontorova model, solitons, yield stress

## Abstract

Solitons are proposed as the agents of plastic and viscoelastic deformation in aligned polyethylene. Interactions between straight, parallel molecules are mapped rigorously onto the Frenkel–Kontorova model. It is shown that these molecular interactions distribute an applied load between molecules, with a characteristic transfer length equal to the soliton width. Load transfer leads to the introduction of tensile and compressive solitons at the chain ends to mark the onset of plasticity at a well-defined yield stress, which is much less than the theoretical pull-out stress. Interaction energies between solitons and an equation of motion for solitons are derived. The equation of motion is based on Langevin dynamics and the fluctuation–dissipation theorem and it leads to the rigorous definition of an effective mass for solitons. It forms the basis of a soliton dynamics in direct analogy to dislocation dynamics. Close parallels are drawn between solitons in aligned polymers and dislocations in crystals, including the configurational force on a soliton. The origins of the strain rate and temperature dependencies of the viscoelastic behaviour are discussed in terms of the formation energy of solitons. A failure mechanism is proposed involving soliton condensation under a tensile load.

## 1. Introduction

**Figure 1.**
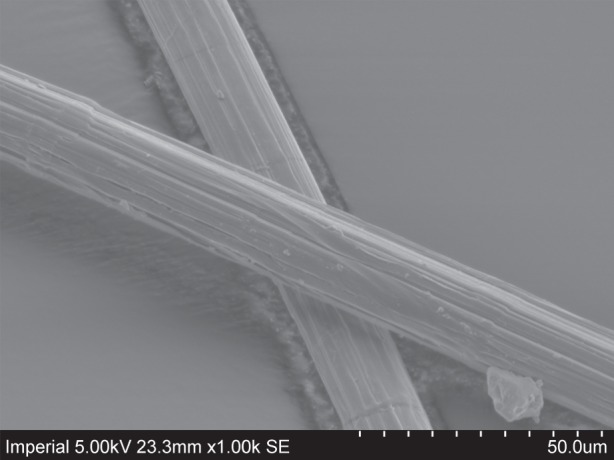
Two Dyneema^®^ SK76 fibres of 17 μm diameter. Notice the cracks parallel to the fibre axis between fibrils. The white markers are 5 μm apart, and the distance between the far left and right markers is 50 μm.

**Figure 2.**
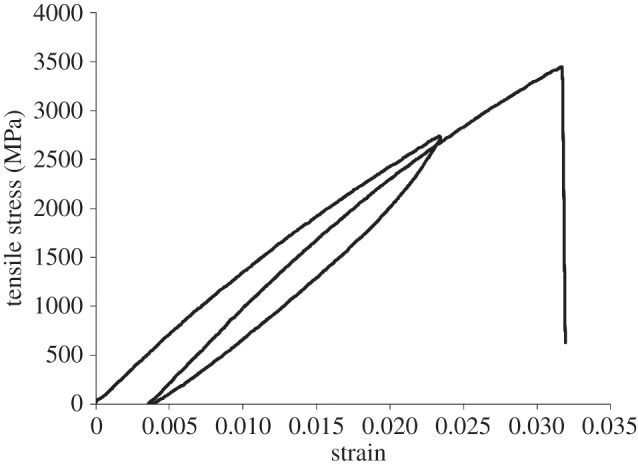
Experimental stress–strain relation for a loading and unloading cycle displaying hysteresis followed by loading to fracture. The material was Dyneema^®^ SK75 yarn comprising 17 μm fibres. The strain rate was 0.001 s^−1^.

Gel-spun fibres of aligned polyethylene are widely used owing to their exceptional specific strength. Experiments have shown that the fibres comprise predominantly highly aligned, long molecular chains, together with a much smaller volume fraction of amorphous regions [1]. Figure 1 shows a scanning electron microscope image of two Dyneema^®^ fibres of 17 μm diameter. Each fibre comprises a bundle of fibrils, which in turn comprise millions of aligned polyethylene molecules. Under a tensile load the material deforms viscoelastically. For example, figure 2 shows stress–strain relations for Dyneema^®^ yarn comprising fibres of 17 μm diameter subjected to a cycle of loading and unloading followed by reloading to fracture at about 3.5 GPa. A hysteresis between the loading and unloading cycle is clearly visible. The magnitude of the hysteresis between loading and unloading decreases with increasing strain rate and decreasing temperature. Time-dependent stress relaxation and creep are also observed. Examining the first deformation cycle in figure 2, the length of the fibre at the end of unloading is greater than at the start of the first loading. After a period of 1–2 days at room temperature the fibre is found to contract to its original length. These properties have also been reported in the work of Van der Werff & Pennings [2].

The behaviour illustrated in figure 2 is observed across a range of aligned polymers. It has been fitted to a standard linear solid model, or its generalization the Maxwell–Wiechert model [3]. In this model, stiffnesses of springs and the viscosities of one or more dashpots are fitted to reproduce the observed hysteresis at a particular strain rate. Once fitted, this empirical model is remarkably successful at predicting the mechanical response of the material at different strain rates [4], but there is no attempt to capture the molecular mechanisms responsible for the mechanical response of the material.

We propose a molecular mechanism of plastic deformation of aligned polymers which is analogous to dislocations in bulk crystals. The mechanism follows from the following observation: while there is no *long* range order in the arrangement of the parallel molecular chains, in contrast to the long-range intermolecular order displayed by crystalline polyethylene, nevertheless there is *short*-range order. The origin of the short-range order is repulsion between molecules at very small separations and van der Waals attraction between molecules at larger separations. Together these interactions favour (i) particular molecular spacings and (ii) certain relative translations of the molecules along the molecular axis. The intermolecular short-range order, coupled with the long-range intramolecular order, leads to the existence of solitons. We contend these solitons are the agents of plastic deformation of aligned polyethylene, and we show their relationship to dislocations in crystalline solids. The existence of solitons in *crystalline* polyethylene has been extensively discussed in the literature, but this has been in the context of dielectric relaxation, not mechanical properties [5–9].

We illustrate these ideas with the simplest possible model of polyethylene, namely a bead-spring model in which each bead represents one CH_2_−CH_2_ monomer. The simplicity of the model is such that many interesting and useful results may be derived analytically. In a subsequent paper [10], we will consider a more realistic model of the structure of polyethylene, which captures the zig-zag structure of the backbone and the torsional degrees of freedom displayed by each polymer chain.

Beads on parallel chains are assumed to interact through a Lennard–Jones (LJ) potential that describes the short-range repulsion and the long-range van der Waals attraction. We show analytically that in the ground state two adjacent chains have a staggered relative translation and a preferred separation. If a finite chain is stretched in the presence of another unloaded chain the deformation is initially elastic and load is transferred between them. We show that the ‘transfer length’ may be derived in a very similar way to that in a fibre-reinforced composite. Stretching the chain further results in plastic deformation where solitons are created, in which the stretch is localized into a relatively small region of the stretched chain, putting the other chain into a localized region of compression. We call this a soliton pair, and it is closely related to an edge dislocation. This result is generalized analytically to the case of a stretched chain surrounded by six other chains in a hexagonal arrangement. The introduction of solitons marks the transition from elastic to plastic behaviour and we derive an equation for the yield stress, in which the yield stress is inversely proportional to the square root of the chain length. By contrast, theoretical chain pull-out, in which a molecular chain is pulled out rigidly *en masse*, is associated with much higher stresses that increase linearly with the length of the aligned segments.

Interaction energies between solitons on the same chain are derived analytically, and the nonlinearity of the sine-Gordon equation leads to interaction energies between solitons of the same and opposite signs that differ at short range in magnitude as well as sign. Finally, in this paper, we derive an equation of motion for the solitons based on the Langevin equation and the fluctuation–dissipation theorem in which an effective mass of the soliton is derived.

The ideas presented in this paper form the basis of a theory of the mechanical properties of aligned polymers based on a molecular mechanism of plastic deformation. Mechanisms underlying viscoelastic properties are readily envisaged involving groups of solitons piled up at barriers to their motion. We show that solitons on adjacent chains attract each other and may form clusters, like the condensation of vacancies in a crystal into prismatic loops. Under the action of a tensile load further solitons may be attracted into the cluster and nucleate a mode I crack. There is a large programme of work involved in developing these ideas through computer simulations with realistic models, like those in [10]. The aim here is to lay the conceptual foundations for this programme of computational research.

## 2. Straight chain model for aligned polyethylene molecules

**Figure 3.**
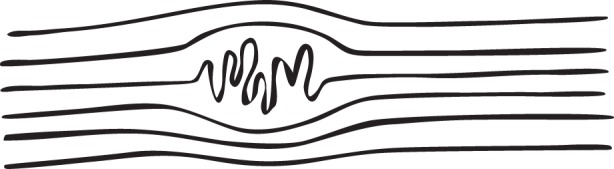
Sketch of the molecular structure assumed in this paper for aligned polyethylene. Most of the material is assumed to consist of parallel molecules with around 5% amorphous regions.

We will show that when a soliton is created at one end of a straight segment, and moves along the straight segment, vanishing at the other end, the entire straight segment is translated by one monomer parallel to itself. We may view the amorphous regions bounding the straight segments as reservoirs, supplying or absorbing monomers to allow the straight segment to be translated parallel to itself when a soliton passes along it (see figure 3). It is not necessary to treat the amorphous regions explicitly and instead we replace them with space. Our model then comprises straight segments of finite length separated by gaps.

In the straight chain model each CH_2_−CH_2_ monomer is replaced by a single bead. The beads are connected by harmonic springs with a spring constant, *κ*, that captures both the C−C bond stiffness and the C−C−C bond bending stiffness. In this way, the polyethylene molecule is represented by a straight linear chain of beads connected by springs.

Beads on different chains, and beads on the same chain that are not directly bonded to each other, are assumed to interact through the following LJ potential to describe the short-range repulsion and the long-range van der Waals attraction:

2.1 $$V_{\mathrm{LJ}}=\frac{1}{2}\sum_{i,j(|i-j|>1)}4\epsilon \left[{\left(\frac{\sigma }{r_{ij}}\right)}^{12}-{\left(\frac{\sigma }{r_{ij}}\right)}^{6}\right],$$

where *r*_*ij*_ is the separation between bead *i* and bead *j*, *ϵ* is an energy parameter and *σ* is a length parameter. The sum over *i* and *j* is taken over all beads in all chains, but the exclusion of self-interactions and interactions between directly bonded beads is indicated by |*i*−*j*|>1. Beads on the same chain are included in the sum over *i* and *j* to ensure that the two ends of a chain repel each other if periodic boundary conditions are employed in simulations. Table 1 shows the parameters used in the LJ potential, and they are taken from [11,12].^1^

**Table 1.** Parameters for the straight chain model.

parameter	value	reference
*ϵ*	0.01036 eV	[11]
*σ*	3.8 Å	[11]
*b*	1.52 Å	[11]
*θ*_0_	1.990 rads	[12]
*k*_*b*_	39.03 eV Å^−2^	[12]
*k*_*θ*_	6.454 eV	[12]

Harmonic springs are used to model the elastic stiffness of the polyethylene backbone. The two independent modes of extension that contribute to the spring constant *κ* are changes *b*→*b*+Δ*b* in the length of the C−C bonds and changes *θ*→*θ*+Δ*θ* of the C−C−C bond angles. The energy associated with the torsion angle of C−C−C−C dihedrals is not included in this model.

Within each monomer there are two C−C bonds and two C−C−C bond angles. The energies associated with bond stretching and bond bending in each monomer unit resulting from a uniform stretch of the entire polymer are

2.2 $$V_{\mathrm{stretch}}=k_{b}{\left(\Delta b\right)}^{2}$$

and

2.3 $$V_{\mathrm{bend}}=k_{\theta }{\left[\cos(\theta_{0}+\Delta \theta )-\cos(\theta_{0})\right]}^{2},$$

where *k*_*b*_ is the stiffness of a single C−C bond, *k*_*θ*_ is the stiffness associated with changing a single C−C−C bond angle and *θ*_0_ is the ideal bond angle. The total elastic energy per monomer of a uniformly stretched chain is *V*_stretch_+*V*_bend_.

By considering a small uniform stretch of the straight chain it is straightforward to show that the effective stiffness *κ* of each spring in the straight chain is as follows:

2.4 $$\kappa =\frac{8k_{b}k_{\theta }\sin^{2}(\theta_{0}/2)}{k_{b}b^{2}+16k_{\theta }\sin^{4}(\theta_{0}/2)}.$$

Using the parameters given in table 1, we find *κ* is 10.034 eV Å ^−2^ (160.76 Nm^−1^) and the ideal spacing *a* of the beads is $2b\sin(\theta_{0}/2)=2.550\;\text{Å}$ (0.2550 nm). The elastic energy associated with each spring in the straight chain model is then $\frac{1}{2}\kappa {\left(\delta a\right)}^{2}$, where *δa* is the deviation in length of each spring from the ideal value *a*.

## 3. The Frenkel–Kontorova model

### (a) Frenkel–Kontorova model for two interacting straight chains

The interaction energy between two straight chains can be represented by a modified version of the Frenkel–Kontorova (FK) model. The FK model describes the energy of a chain of beads connected by harmonic springs in the field of a *rigid* periodic potential [13,14]. The ideal spacing of the beads in the chain is *a* and is equal to the period of the potential. In the ground state, each bead is sitting in a minimum of the periodic potential, and no bonds are stretched. For a single molecular chain in the presence of the rigid periodic potential, the energy according to the FK model is

3.1 $$E=\sum_{i}\frac{\kappa }{2}{\left(u_{i+1}-u_{i}\right)}^{2}+\sum_{i}V\sin^{2}\left(\frac{\pi u_{i}}{a}\right),$$

where *u*_*i*_ is the displacement of bead *i* from its equilibrium position and *V* is twice the amplitude of the periodic potential. The FK model predicts the formation of a soliton when the chain is stretched by *a* relative to the fixed periodic potential [13,14]. The soliton is a localization of the applied strain, and its width is proportional to $\sqrt{\kappa a^{2}/V}$.

**Figure 4.**
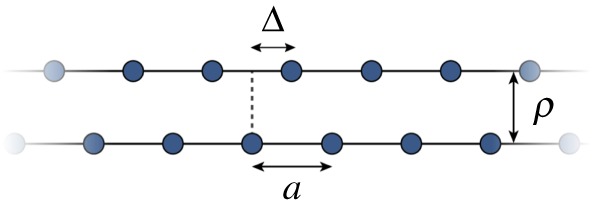
Two infinite, parallel, straight chains, separated by *ρ*, with a rigid relative translation parallel to the chains of Δ. The spacing of the beads in each chain is *a*.

To show that solitons may arise in aligned polyethylene, we first demonstrate that the periodic potential of the FK model can be mapped onto the potential of a single infinite chain (figure 4). As the LJ potential is pairwise additive, it is sufficient to consider the energy of interaction between one bead of a chain with all the beads of another chain. This is done by positioning a single interacting bead at a distance *ρ* from the chain, moving that bead parallel to the chain and finding an analytic expression for the interaction energy between the bead and the chain. We will show that this interaction energy, *E*_LJ_, can be approximated very well by a periodic potential of the form $C+V\sin^{2}(\pi \Delta /a)$, where *C* is a constant and Δ is the relative displacement of the chains from the configuration where the beads are directly opposite each other. In this way, each chain provides a periodic potential that can be felt by another chain in its proximity. *E*_LJ_ comprises a sum of repulsive interaction energies *E*_rep_ and attractive interaction energies *E*_att_, which can be expressed as follows:

3.2 $$\begin{array}{rl} E_{\mathrm{att}}(\Delta ) & =-4\epsilon \sum_{n=-\infty }^{\infty }\frac{\sigma^{6}}{{\left[\rho^{2}+{\left(na+\Delta \right)}^{2}\right]}^{3}}\\ & =b_{0}+\sum_{p=1}^{\infty }b_{p}\cos\left(\frac{2\pi p\Delta }{a}\right) \end{array}$$

and

3.3 $$\begin{array}{rl} E_{\mathrm{rep}}(\Delta ) & =4\epsilon \sum_{n=-\infty }^{\infty }\frac{\sigma^{12}}{{\left[\rho^{2}+{\left(na+\Delta \right)}^{2}\right]}^{6}}\\ & =c_{0}+\sum_{p=1}^{\infty }c_{p}\cos\left(\frac{2\pi p\Delta }{a}\right), \end{array}$$

where (*b*_0_,*b*_*p*_) and (*c*_0_,*c*_*p*_) are Fourier coefficients. In the electronic supplementary material, we show the interaction energy per bead is approximated very well by

3.4 $$E_{\mathrm{LJ}}(\Delta )=-28.2-1.035\sin^{2}\left(\frac{\pi \Delta }{a}\right)\mathrm{meV}.$$

It is also shown in the electronic supplementary material that the equilibrium separation of the chains is *ρ*=4.124 Å and the energy of interaction between the chains at this separation is −0.029 eV *a*^−1^. It follows from equation (3.4) that the interaction energy is minimized when Δ=*a*/2, that is the ground-state configuration of the chains is staggered.

Consider a straight molecular segment of length *L* at the centre of a hexagon of six straight molecules of length at least *L*. The molecules are at the equilibrium separation *ρ*=4.124 Å . The cross-sectional area associated with the central molecule is $\sqrt{3}\rho^{2}/2$. By differentiating equation (3.4), we may estimate the theoretical tensile stress required to pull-out the central molecule as a rigid object as follows:

3.5 $$\begin{array}{rl} \sigma_{\mathrm{pull}\text{-}out} & =6N_{M}\cdot {\left|\frac{dE_{\mathrm{LJ}}}{d\Delta }\right|}_{\Delta =a/4}\cdot \frac{2}{\sqrt{3}\rho^{2}}\\ & =1.39\times 10^{7}N_{M}\;\mathrm{Pascals}, \end{array}$$

where *N*_M_ =*L*/*a* is the number of monomers in the molecule and the factor of 6 is because the molecule has six neighbours. For example, for a molecule of 10 μm length we obtain *σ*_pull-out_=545 GPa. Thus, although the van der Waals interactions are weak compared with the covalent bonds the very large number (≈40 000) of the former in a molecule of 10 μm length prevents the theoretical pull-out *en masse* of single molecules as rigid objects as a mechanism of plastic deformation.

### (b) Solitons in two interacting chains

Consider two infinitely long interacting chains in the staggered ground state configuration parallel to the *x*-axis. The chain ends at x=−∞ are anchored in position. One chain is stretched uniformly by *a*/2, and the other is compressed uniformly by *a*/2, such that at x=+∞ the chains are again in the staggered ground state. Each chain is then relaxed subject to the boundary conditions that the relative displacements between corresponding beads in the chains are zero at x=−∞ and *a* at x=+∞. If there were no interactions between the chains the tensile and compressive strains would remain uniform along the chains and all bonds would suffer the same infinitesimally small changes of length. We will find that the interaction between the chains leads to a spatial localization of the strains to form tensile and compressive solitons.

There is a crucial difference between this model and the standard FK model. The standard FK model considers the interaction of a single chain with a *rigid* periodic potential, whereas here we treat the interaction between two chains in which the configuration, and hence the potential, of either chain can *vary* to minimize the total energy. We make a *local approximation* to equation (3.4) where the constant relative displacement Δ of the two chains is replaced by the local relative displacement *u*_*i*_−*v*_*i*_. Here *u*_*i*_ and *v*_*i*_ are the displacements of bead *i* in the stretched and compressed chains, respectively, from their positions in the ground state staggered configuration of unstrained chains. The energy of interaction depends on the difference *u*_*i*_−*v*_*i*_ because equal displacements of the two chains from the ground state configuration have no effect on the interaction energy. However, any relative displacement of the two chains always contributes non-negatively to the energy of interaction because it is with respect to the ground state configuration. The local approximation to equation (3.4) is valid provided the relative displacement of the two chains changes slowly. We will see that this condition is satisfied.

The total energy of the two chain system according to the modified FK model is as follows:

3.6 $$E=\sum_{i}\frac{\kappa }{2}{\left(u_{i+1}-u_{i}\right)}^{2}+\sum_{i}\frac{\kappa }{2}{\left(v_{i+1}-v_{i}\right)}^{2}+\sum_{i}V\sin^{2}\left(\frac{\pi (u_{i}-v_{i})}{a}\right).$$

The first two terms are the elastic energy in the stretched and compressed chain, while the third is the interaction energy between the two chains. The constant energy on the right of equation (3.4) is dropped because it makes no difference to the displacements.

Provided the displacement fields *u*_*i*_ and *v*_*i*_ are slowly varying, we may take the continuum limit in the usual way. In that limit $\sum_{i}\to \int (dx/a)$, *u*_*i*_→*u*(*x*), *u*_*i*+1_→*u*(*x*+*a*)→*u*(*x*)+*a* d*u*/d*x* and similarly for *v*. We can then write equation (3.6) in the continuum limit as follows:

3.7 $$E=\frac{\kappa a^{2}}{2}\int_{-\infty }^{\infty }\frac{dx}{a}\left[{\left(\frac{du}{dx}\right)}^{2}+{\left(\frac{dv}{dx}\right)}^{2}\right]+V\int_{-\infty }^{\infty }\frac{dx}{a}\sin^{2}\left(\frac{\pi (u-v)}{a}\right).$$

The corresponding coupled Euler–Lagrange equations are

3.8 $$\kappa a\frac{d^{2}u}{dx^{2}}=\frac{\pi V}{a^{2}}\sin\left(\frac{2\pi (u-v)}{a}\right)$$

and

3.9 $$\kappa a\frac{d^{2}v}{dx^{2}}=-\frac{\pi V}{a^{2}}\sin\left(\frac{2\pi (u-v)}{a}\right).$$

By subtracting and adding equations (3.8) and (3.9) we obtain

3.10 $$\frac{d^{2}(u-v)}{dx^{2}}=\frac{2V\pi }{\kappa a^{3}}\sin\left(\frac{2\pi (u-v)}{a}\right)$$

and

3.11 $$\frac{d^{2}(u+v)}{dx^{2}}=0.$$

Equation (3.10) is the time-independent sine-Gordon equation. Equation (3.11) and the boundary conditions at x=±∞ lead to the conclusion that *u*+*v*=0 for all *x*. It then follows from equation (3.10) that

3.12 $$\frac{d^{2}u}{dx^{2}}=\frac{\pi V}{\kappa a^{3}}\sin\left(\frac{4\pi u}{a}\right).$$

The solution to this equation satisfying the boundary conditions on *u* at ±∞ is as follows:

3.13 $$u=\frac{a}{\pi }\tan^{-1}\exp\left(\sqrt{\frac{V}{\kappa a^{2}}}\frac{2\pi x}{a}\right).$$

**Figure 5.**
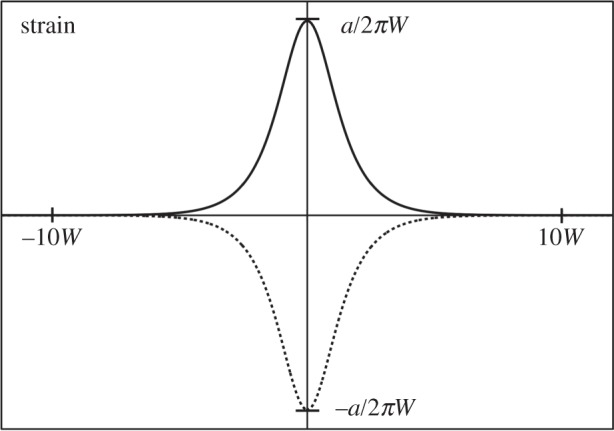
Plots of the strains in a pair of interacting chains stretched (solid line) and compressed (broken line) by *a*/2. The strains are d*u*/d*x*=(*a*/2*πW*)sech (*x*/*W*) and d*v*/d*x*=−(*a*/2*πW*)sech (*x*/*W*), respectively. The strain localization leads to a soliton pair at the origin. The separation of beads along each chain is *a*, and *W* is a measure of the width of the soliton pair along each chain.

Thus, the tensile and compressive strains in the two chains are localized around the origin forming tensile and compressive solitons, as illustrated in figure 5. We may define the width, *W*, of the solitons as follows:

3.14 $$W=\frac{a}{2\pi }\sqrt{\frac{\kappa a^{2}}{V}.}$$

For the parameters in table 1, we find *W*≈100 Å ≈40*a*.

### (c) Solitons in a hexagonal array of aligned polyethylene molecules

In this section, we consider a central chain subjected to a tensile strain and interacting with six surrounding chains in a hexagonal arrangement. Each of the surrounding chains is initially in the ground state staggered configuration with respect to the central chain. The six surrounding chains are equivalent and undergo the same distortions.

Neglecting all interactions beyond first neighbours, the spacing of the molecules in a hexagonal arrangement is *ρ*=4.124 Å and each bead is associated with an interaction energy with its six neighbouring chains of 0.086 eV, equivalent to an interaction energy per unit length of each molecule of 0.034 eV Å ^−1^=0.054 nJ m^−1^. These interactions lead to a cohesive energy of 370 MJ m^−3^, defined as the energy required to separate 1 m^3^ of hexagonally packed, aligned polyethylene molecules into isolated polyethylene molecules. For comparison this is approximately 0.2% of the cohesive energy of diamond [15], highlighting the huge difference between van der Waals and covalent bonding. The weakness of the intermolecular forces, in comparison to the intramolecular forces, is evident in figure 1 where cracks are evident parallel to the fibre axis, which have been found to have no significant influence on the ultimate tensile strength of the fibres [16].

At this value of the intermolecular separation, *ρ*=4.124*a*, a hexagonal array of perfect polyethylene molecules, i.e. with no amorphous regions, at absolute zero would have a density of 1.24 g cm^−3^, and Young's modulus would be 2.78×10^11^ Pa. If the effect of amorphous regions is to reduce the density by increasing the average spacing between hexagonally arranged molecular chains, then the relationship between Young's modulus *Y* , expressed in GPa and the density *D*, expressed in g cm^−3^, is *Y* =225*D*. The influence of a finite temperature will be to reduce the density further through thermal expansion.

Let *u*_*i*_ be the displacement of bead *i* in the central chain. Let *v*_*i*_ be the displacement of bead *i* in any one of the six neighbouring chains. The boundary conditions are $u_{-\infty }=0,\;v_{-\infty }=0$, and $u_{\infty }-v_{\infty }=a$. The total energy of the seven chains is as follows:

3.15 $$E=\sum_{i=-\infty }^{\infty }\frac{\kappa }{2}{\left(u_{i+1}-u_{i}\right)}^{2}+\frac{6\kappa }{2}{\left(v_{i+1}-v_{i}\right)}^{2}+6V\sin^{2}\left(\frac{\pi (u_{i}-v_{i})}{a}\right).$$

Taking the continuum limit as before, the total energy becomes

3.16 $$E=\int_{-\infty }^{\infty }\frac{dx}{a}\left[\frac{\kappa a^{2}}{2}{\left(\frac{du}{dx}\right)}^{2}+\frac{6\kappa a^{2}}{2}{\left(\frac{dv}{dx}\right)}^{2}+6V\sin^{2}\left(\frac{\pi (u-v)}{a}\right)\right].$$

The corresponding Euler–Lagrange equations are

3.17 $$\frac{d^{2}u}{dx^{2}}=\frac{6\pi V}{\kappa a^{3}}\sin\left(\frac{2\pi (u-v)}{a}\right)$$

and

3.18 $$\frac{d^{2}v}{dx^{2}}=-\frac{\pi V}{\kappa a^{3}}\sin\left(\frac{2\pi (u-v)}{a}\right).$$

Adding six times equation (3.18) to equation (3.17), we deduce that d^2^(*u*+6*v*)/d*x*^2^=0. It follows from this and the boundary condition at x=−∞ that *u*=−6*v* at all *x*. Hence the boundary condition at x=+∞ is satisfied if u(∞)=6a/7 and v(∞)=−a/7. Subtracting equations (3.17) and (3.18) we obtain

3.19 $$\frac{d^{2}(u-v)}{dx^{2}}=\frac{7\pi V}{\kappa a^{3}}\sin\left(\frac{2\pi (u-v)}{a}\right).$$

The solutions for *u*(*x*) and *v*(*x*) are

3.20 $$u(x)=\frac{12a}{7\pi }\tan^{-1}\left[\exp\left(\sqrt{\frac{14V}{\kappa a^{2}}}\frac{\pi x}{a}\right)\right]$$

and

3.21 $$v(x)=-\frac{2a}{7\pi }\tan^{-1}\left[\exp\left(\sqrt{\frac{14V}{\kappa a^{2}}}\frac{\pi x}{a}\right)\right].$$

It is evident that the width, *W*_6_≈55 Å ≈21*a*, of the solitons in this case is a factor of $\sqrt{\frac{7}{2}}$ narrower than in the case of two interacting chains. This is still ample to justify the continuum approximation for the displacement fields and the local approximation for the interaction energy.

**Figure 6.**
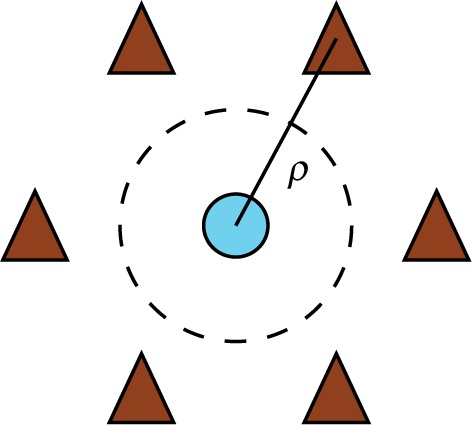
Prismatic dislocation loop (broken circle) formed by an extensive soliton in the inner chain (blue circle) and compressive solitons in the six outer chains (brown triangles). The chains are viewed in projection along their axes.

The tensile strain in the central chain and the compressive strains in the six surrounding chains suggests an equivalence to a vacancy prismatic dislocation loop. The Burgers vector of the loop has magnitude *a*, and its direction is parallel to the chain axis. The dislocation line is a loop surrounding the central chain and passing between it and the six surrounding chains, as shown by the broken line in figure 6. Indeed Dudarev [17] has shown a formal equivalence between clusters of crowdions in a metal and a dislocation loop using the FK model. Viewing solitons in aligned polymers as dislocation loops enables one to draw conceptual connections between the plastic properties of aligned polymers and those of crystalline metals.

## 4. Load transfer and the yield stress

Consider a finite chain of length *L* subjected to a tensile force *F* at its ends. Let the displacement of bead *i* in this chain be *u*_*i*_. Let this chain be parallel to an unloaded chain of at least the same length in which the displacement of bead *i* is *v*_*i*_. The chains are at the equilibrium separation.

Equations (3.8) and (3.9) again define the displacement fields *u*(*x*) and *v*(*x*) in the continuum approximation. It is convenient to choose *x*=0 at the centre of the loaded chain. The unloaded chain experiences no force at |*x*|>*L*/2: in these regions *v*(*x*)=0. But for |*x*|<*L*/2 the interaction energy between the chains gives rise to a force acting on the unloaded chain, which results in transfer of some of the force in the loaded chain to the unloaded chain. This is what is meant by load transfer.

The boundary conditions for *u*(*x*) and *v*(*x*) are

4.1 $$\begin{array}{rl} {\left(\frac{du}{dx}\right)}_{x=\pm L/2} & =\frac{F}{\kappa a}\\ \;\text{and}\;{\left(\frac{dv}{dx}\right)}_{x=\pm L/2} & =0. \end{array}\}$$

Adding equations (3.8) and (3.9) and using the boundary conditions at *x*=±*L*/2 it can be shown that in |*x*|≤*L*/2 we have *u*(*x*)+*v*(*x*)=*Fx*/(*κa*). Setting *w*(*x*)=*u*(*x*)−*v*(*x*) and subtracting equations (3.8) and (3.9), we obtain $\kappa a\;d^{2}w/dx^{2}=(2\pi V/a^{2})\sin(2\pi w/a)$. In the *elastic* regime *w*(*x*)≪*a*, we then obtain

4.2 $$\frac{d^{2}w}{dx^{2}}=\frac{w}{W^{2}},$$

where *W* is the soliton width in the two-chain system, defined in equation (3.14). Noting that symmetry requires *w*(*x*) to be an odd function and using the boundary condition d*w*/d*x*=*F*/(*κa*) at *x*=±*L*/2, the solution for *w*(*x*) is

4.3 $$w(x)=\frac{F}{\kappa a}W\frac{\sinh(x/W)}{\cosh(L/2W)}.$$

It follows that the forces carried by the two chains are as follows:

4.4 $$\begin{array}{rl} \kappa a\frac{du}{dx} & =\frac{F}{2}\left(1+\frac{\cosh(x/W)}{\cosh(L/2W)}\right)\\ \;\text{and}\;\kappa a\frac{dv}{dx} & =\frac{F}{2}\left(1-\frac{\cosh(x/W)}{\cosh(L/2W)}\right). \end{array}\}$$

**Figure 7.**
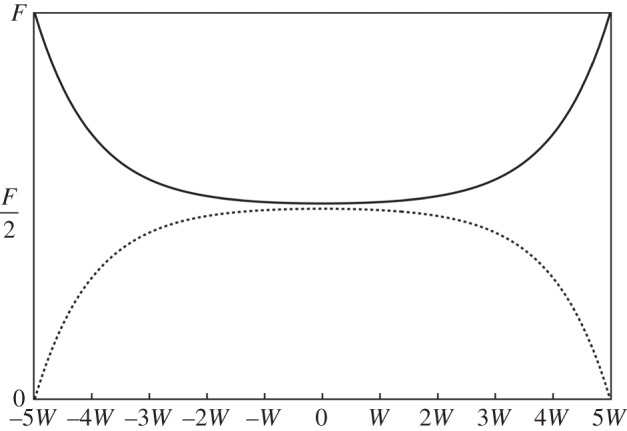
The load carried by the directly loaded chain (solid line) and the load transferred (broken line) to a second interacting chain in two chains of length *L*=10*W*. The horizontal axis is *x* in equations (4.4). Note that at any position along the chains the total load carried by both is *F*.

Equations (4.4) are plotted in figure 7 for chains of length 10*W*.

The average load transfer to the chain that was not directly loaded is given by

4.5 $$\frac{\kappa a}{L}\int_{0}^{L}\frac{dv}{dx}dx=\frac{F}{2}\left(1-\left(\frac{2W}{L}\right)\tanh\left(\frac{L}{2W}\right)\right).$$

When *W*≪*L* the average load transfer is the maximum, *F*/2. When 2*W*≈*L* the average load transfer decreases to about a quarter of the maximum. It is evident that *W* is not only a measure of the soliton width but it also characterizes the length over which load is effectively transferred between chains. That is, unless the chains are longer than ≈5*W* the load transfer is ineffective. At first sight it may be surprising that the transfer length and the soliton width are the same. But they are defined by the sine-Gordon equation, albeit in different limits, and there is only one length scale, *W*, in that equation.

The elastic energy stored in the two chains when the directly loaded chain is subjected to a tensile force *F* at its ends is

4.6 $$E_{\mathrm{el}}=\frac{F^{2}L}{4\kappa a}\left(1+\left(\frac{2W}{L}\right)\tanh\left(\frac{L}{2W}\right)\right).$$

In the limit *L*≫*W* this becomes *E*_el_=*F*^2^*L*/(4*κa*). This is just the elastic energy stored in two chains of length *L*, each subjected to a tensile force *F*/2, as expected when the load is distributed equally between the two chains. Thus, for a given applied force *F* and *L*≫*W* the stored elastic energy is proportional to the chain length. The energy of a soliton pair in an infinite pair of chains is $E_{1}=(2/\pi )\sqrt{\kappa a^{2}V}=0.165\;\mathrm{eV}$. This is also a very good approximation to the energy of a soliton pair in chains of finite length when *L*≫*W*.

It becomes energetically favourable to introduce a soliton pair when the following condition is satisfied:

4.7 $$\frac{F}{\kappa a}\geq \frac{2}{\pi }\sqrt{\frac{a^{2}}{WL}}.$$

This is a necessary but not sufficient condition to introduce a soliton because there has to be a mechanism to introduce it. It follows that the minimum applied force *F* necessary for the introduction of a soliton is inversely proportional to the square root of the chain length. For *W*≈40*a* and *L*=10^3^*W*≈10 μm, we obtain *F*≈0.013 eV Å ^−1^≈0.021 nN. One place where solitons may be introduced readily is a chain end, where the relative displacement, *w*(*x*)=*u*(*x*)−*v*(*x*) given in equation (4.3), of the chains is maximized. This has been confirmed by simulations and will be reported in detail in a subsequent paper [10].

We may repeat this argument for a molecule at the centre of a hexagon of other molecules. The energy of forming a soliton in this case is given by inserting equations (3.20) and (3.21) for *u*(*x*) and *v*(*x*) into equation (3.16), which yields $(24/\pi )\sqrt{\kappa a^{2}V/14}=0.529\;\mathrm{eV}$. For the calculation of the elastic energy before yielding we assume again that the molecular length *L* is much larger than the transfer length. In that case the load transfer to the six surrounding molecules is maximized. The central molecule will then bear half the applied load *F*, while each of the surrounding six molecules will bear *F*/12. The total elastic energy stored in the seven chains is then *F*^2^*L*/(6*κa*). By dividing the solution for *F* defined by $F^{2}L/(6\kappa a)=(24/\pi )\sqrt{\kappa a^{2}V/14}$ by the area $\left(\sqrt{3}/2\right)\rho^{2}$ associated with the central chain we obtain the following expression for the yield stress, *σ*_*y*_:

4.8 $$\sigma_{y}=\sqrt{\frac{96a^{2}}{7W_{6}L}}\frac{\kappa a}{\pi \rho^{2}},$$

where $W_{6}=(a/\pi )\sqrt{\kappa a^{2}/(14V)}$ is the soliton width in the hexagonal cluster. For *L*=10 μm the predicted yield stress is 310 MPa.

It follows that the introduction of solitons occurs at stresses orders of magnitude less than those required for theoretical chain pull-out. In addition, while the stress required for theoretical pull-out is proportional to the length, *L*, of the molecule, the stress required to introduce solitons is proportional to *L*^−1/2^. An increase in the density of the sample, brought about by a reduction in the average spacing *ρ* of molecules, will increase the yield stress. Equation (4.8) is quite remarkable because it expresses the yield stress of the material in terms of quantities that can be related back to the fundamental molecular parameters in table 1, together with the length *L* of straight segments of the molecules.

## 5. Interaction energy between solitons

In this section, we consider the interaction energy between two soliton pairs of the same polarity and of opposite polarity in the two-chain model. We explain what we mean by ‘polarity’ below. Unlike dislocations these interaction energies differ at short range in magnitude as well as sign.

In the two-chain model with *v*(*x*)=−*u*(*x*) equation (3.7) gives the following energy functional for a soliton pair in two infinite chains:

5.1 $$E[u(x)]=\kappa a^{2}\int_{-\infty }^{\infty }\frac{dx}{a}{\left(\frac{du}{dx}\right)}^{2}+V\int_{-\infty }^{\infty }\frac{dx}{a}\sin^{2}\left(\frac{2\pi u}{a}\right).$$

Let *u*_1_(*x*) and *u*_2_(*x*) be the solutions for two tensile solitons at *x*=0 and *x*=*X*, respectively, in one chain. There are then two compressive solitons at *x*=0 and *x*=*X* in the other chain. In this case, we say the soliton pairs have the same polarity because on each chain the solitons are either both tensile or both compressive. The soliton pairs have opposite polarity when one of the solitons on each chain is tensile and the other compressive. Equations (3.13) and (3.14) give the following solutions for *u*_1_(*x*) and *u*_2_(*x*):

5.2 $$u_{1}(x)=\frac{a}{\pi }\tan^{-1}\exp\left(\frac{x}{W}\right)$$

and

5.3 $$u_{2}(x)=\frac{a}{\pi }\tan^{-1}\exp\left(\frac{\left(x-X\right)}{W}\right).$$

The interaction energy between soliton pairs is defined by

5.4 $$E_{\mathrm{int}}=E[u_{1}+u_{2}]-E[u_{1}]-E[u_{2}].$$

**Figure 8.**
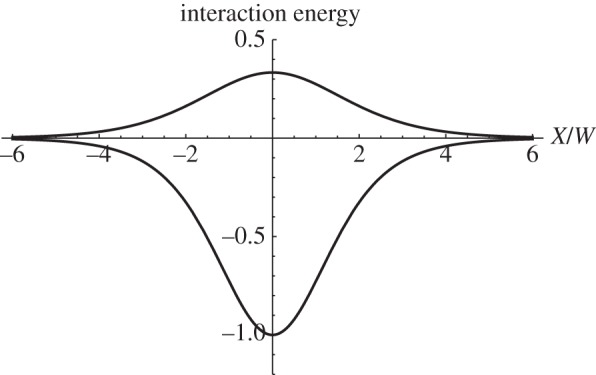
Interaction energy, in units of 2*E*_1_, for two soliton pairs of the same (upper curve) and opposite polarities (lower curve), as a function of their separation $X/W=\bar{X}$, as given by equations (5.5) and (5.6), respectively.

The evaluation of the integrals in equation (5.4) is tedious and here we state the results. More details are given in the electronic supplementary material. For two soliton pairs of the same polarity the interaction energy is as follows:

5.5 $$E_{\mathrm{int}}^{↑↑}=E_{\mathrm{int}}^{↓↓}=\frac{2E_{1}}{\sinh^{3}\bar{X}}(\sinh\bar{X}-\bar{X})(\cosh\bar{X}+1),$$

where $\bar{X}=X/W$ and an up/down arrow indicates the polarity of a soliton pair. The energy of an isolated soliton pair is again *E*_1_. As seen in figure 8, the interaction energy is positive at all separations so that the soliton pairs repel each other. In the limit *X*→0, the interaction energy →2*E*_1_/3. Thus the total energy of two superimposed soliton pairs of the same polarity is finite and equal to 8*E*_1_/3. As X→±∞ the interaction energy decays to zero as $4E_{1}\exp(-|\bar{X}|)$.

For two soliton pairs of opposite polarity the interaction energy is as follows:

5.6 $$E_{\mathrm{int}}^{↑↓}=E_{\mathrm{int}}^{↓↑}=-\frac{2E_{1}}{\sinh^{3}\bar{X}}(\sinh\bar{X}+\bar{X})(\cosh\bar{X}-1).$$

As seen in figure 8, the interaction is attractive at all separations and as *X*→0 the interaction energy becomes −2*E*_1_ and the total energy becomes zero: the soliton pairs annihilate each other. As X→±∞ the interaction energy decays as $-4E_{1}\exp(-|\bar{X}|)$. It is striking that at small separations, compared with the soliton width *W*, the interaction energies in equations (5.5) and (5.6) do not differ merely in sign, and this is evident in figure 8. This interesting result is a consequence of the nonlinearity of the sine-Gordon equation. Finally, the force exerted by one soliton pair on another is obtained by differentiating equations (5.5) and (5.6) with respect to *X*. This force can be used in an equation of motion for solitons, as shown in the next section.

## 6. Equation of motion

Solitons may move along the polyethylene chains, exert forces of interaction on each other and be created or annihilated. In this section, we derive a Langevin equation of motion for solitons that includes the influence of random thermal forces, atomic-level friction, forces of interaction with other solitons and forces arising from an external load. The objective is to develop a ‘soliton dynamics’ to model plasticity and viscoelasticity of aligned polymers in terms of the dynamics of soliton pairs viewed as quasi-particles, in much the same way as dislocation dynamics is used to model plasticity in crystalline materials. The fluctuation–dissipation theorem relates the frictional force and the random thermal forces in the usual way. One of the central results of this analysis is the derivation of an effective mass of solitons.

The total energy of the soliton pair includes the potential energy arising from the interaction energy with other solitons and with an external load, which we write as 2*ϕ*(*u*) for convenience. It also includes the kinetic energy of the beads in the two chains, so that *u* becomes a function of time *t* as well as *x*. Let *m* be the mass of each bead, which we recall is a monomer. Provided the speed of the soliton is much less than the longitudinal speed of sound along each chain, we may treat the soliton pair as a rigid composite object in which *v*(*x*−*X*(*t*),*t*)=−*u*(*x*−*X*(*t*),*t*), with a centre of mass *X*(*t*):

6.1 $$u(x-X(t))=\frac{a}{\pi }\tan^{-1}\exp\left[\frac{\left(x-X(t)\right)}{W}\right],$$

where *W* is the soliton width defined in equation (3.14). The equation of motion for the soliton pair is then an equation for *X*(t). In the continuum limit, the action becomes

6.2 $$S=\int_{-\infty }^{\infty }dt\int_{-\infty }^{\infty }\frac{dx}{a}\left[m{\left(\frac{\partial u}{\partial t}\right)}^{2}-\kappa a^{2}{\left(\frac{\partial u}{\partial x}\right)}^{2}-V\sin^{2}\left(\frac{2\pi u}{a}\right)-2\phi (u)\right],$$

for which the Euler–Lagrange equation is

6.3 $$m\frac{\partial^{2}u}{\partial t^{2}}=\kappa a^{2}\frac{\partial^{2}u}{\partial x^{2}}-\frac{\pi V}{a}\sin\left(\frac{4\pi u}{a}\right)-\frac{\partial \phi }{\partial u}.$$

In Langevin dynamics, this equation of motion is augmented by a friction force *γ*(∂*u*/∂*t*) and a random force *η*(*x*,*t*) to emulate thermal buffeting. The average value of *η*(*x*,*t*) over time is zero at all *x*, and it is assumed that there is no correlation of the thermal force at different values of *x* and *t*. As is well known the fluctuation–dissipation theorem relates *γ* to the width of the distribution of thermal forces, and the temperature *T*: 〈*η*(*x*,*t*)*η*(*x*′,*t*′)〉=2*γk*_*B*_*Tδ*(*x*−*x*′)*δ*(*t*−*t*′). The equation of motion then becomes

6.4 $$m\frac{\partial^{2}u}{\partial t^{2}}=\kappa a^{2}\frac{\partial^{2}u}{\partial x^{2}}-\frac{\pi V}{a}\sin\left(\frac{4\pi u}{a}\right)-\frac{\partial \phi }{\partial u}+\gamma \frac{\partial u}{\partial t}+\eta (x,t).$$

As *u*=*u*(*x*−*X*(*t*)) we may write $\dot{u}=\dot{X}u^{\prime }$, where the dot denotes differentiation with respect to time and the dash denotes differentiation with respect to the argument of *u*, and $\ddot{u}=\ddot{X}u^{\prime }+{\dot{X}}^{2}u^{\prime \prime }$. Making these substitutions and multiplying both sides of the equation by *u*′, and then integrating over *x* from −∞ to +∞ we obtain

6.5 $$\tilde{m}\ddot{X}=\frac{\tilde{m}}{m}\frac{\partial \phi }{\partial X}+\gamma \dot{X}\frac{\tilde{m}}{m}+\int_{-\infty }^{\infty }\frac{dx}{a}\eta (x,t)u^{\prime },$$

where $\tilde{m}=m\int_{-\infty }^{\infty }(dx/a){\left(u^{\prime }\right)}^{2}=am/(2\pi^{2}W)$ is the effective mass of each soliton in the soliton pair. Defining $f(t)=\int_{-\infty }^{\infty }\left(dx/a)\eta (x,t)u^{\prime }(x\right)$, where 〈*f*(*t*)〉=0 and $\left\langle f(t)f(t^{\prime })\rangle =(2\gamma k_{B}T/a^{2})(\tilde{m}/m)\delta (t-t^{\prime }\right)$ follow from the properties of *η*(*x*,*t*), equation (6.5) becomes

6.6 $$m\ddot{X}=\frac{\partial \phi }{\partial X}+\gamma \dot{X}+\frac{m}{\tilde{m}}\;f(t).$$

Given that $m/\tilde{m}=2\pi^{2}W/a$, and *W*≫*a*, the random thermal force may be expected to dominate the inertial force on the left-hand side. The equation of motion in this overdamped case then becomes

6.7 $$\gamma \dot{X}=-\frac{\partial \phi }{\partial X}+\frac{m}{\tilde{m}}\;f(t).$$

This is the standard equation of motion for solitons interacting with each other, and with an externally applied load, and subjected to random thermal forces [18].

## 7. Discussion

### (a) Solitons in aligned polymers

In this paper, we have argued that the combination of short-range intermolecular order and long-range intramolecular order leads to the existence of solitons in aligned polyethylene. They arise through the relative displacements of adjacent molecular chains which set up forces that localize strains along the molecules. In order for there to be relative displacements between the molecules they have to respond differently to an applied load, and that is always likely to be the case provided they are shorter than the length of the sample over which an external force is applied. Although we have focused on polyethylene the thinking here may be applicable to other aligned molecules such as bundles of aligned carbon nanotubes. Solitons may also arise in aliphatic and aromatic polyamide fibres, e.g. poly *para*-phenyleneterephthalamide (kevlar), but the hydrogen bonding in these systems is significantly stronger than the van der Waals bonding in polyethylene, and it is directional in nature. If they exist in these systems, solitons are likely to have smaller widths and be less mobile than in polyethylene.

### (b) Parallels with dislocations in crystals and a mechanism of fracture

There is a close analogy between the solitons in aligned polymers and dislocations in crystals. In a hexagonal array of polyethylene molecules each tensile soliton is surrounded by six smaller compressive solitons. The seven solitons are bound together as a quasi-particle which may be thought of as a vacancy prismatic dislocation loop (figure 6). Vacancy prismatic loops centred on the same molecule will repel each other. But if they are centred on adjacent molecules they will attract and form a divacancy loop with the tensile and compressive solitons now extending over ten molecules. The process can be repeated on further adjacent molecules so that the dislocation loop encloses more vacancies. Under the influence of an applied tensile load further tensile solitons will be attracted elastically into the vacancy loop and a mode I crack may then be nucleated. This failure mechanism is more likely to operate at low temperatures where interaction energies between solitons can drive the condensation.

Consider a tensile soliton on a molecule of length *L* subjected to a tensile load *F* in a hexagonal array of molecules. As before, we let the separation between molecules be the equilibrium value *ρ*. The soliton introduces a relative displacement between the central molecule and the six surrounding molecules of *a*. As we have seen the tensile soliton in the central chain introduces a compressive soliton in each of the six surrounding chains. We will show that the force on this group of solitons is the same as the Peach–Koehler force acting on the equivalent vacancy prismatic dislocation loop with Burgers vector magnitude equal to *a*. If the soliton group passes from one end of the central molecule to its other end the potential energy of the loading mechanism is decreased by *Fa*. If the soliton group moves a distance *δx*, the potential energy is decreased by *Fa*(*δx*/*L*). Therefore, the force on the soliton group is *Fa*/*L*. But the tensile force *F* generates a shear stress *τ* on a notional cylindrical surface of radius 0<*r*<*ρ* surrounding the central molecule equal to *F*/(2*πrL*). Therefore, the force on the soliton group is *Fa*/*L*=2*πrLτa*/*L*=2*πrτa* which is just the Peach–Koehler force *τa* per unit length acting on a dislocation loop of length 2*πr* and Burgers vector magnitude *a*. Note that the Peach–Koehler force per unit length of dislocation is independent of the radius *r* assumed for the dislocation loop and of the length *L* assumed for the central chain.

The analogy between solitons and vacancy prismatic loops suggests interesting mechanisms that may play a role in the mechanical behaviour of aligned polymers. We have already seen how mode I cracks may be formed through the condensation of soliton groups forming vacancy clusters. In a subsequent paper [10], we will consider defects in a more realistic model of a polyethylene molecule respecting the zig-zag nature of the backbone. One of these defects, the ‘2*π* twiston’, is highly localized and acts as a barrier to the motion of solitons along the molecule. Therefore, a pile up of solitons at a 2*π* twiston is possible and may act as a stress concentrator, in much the same way as a pile of dislocations at a grain boundary or particle concentrates stress in a polycrystal. The existence of such barriers may also explain the Bauschinger effect and the eventual recovery of the initial length of tensile specimens referred to in the introduction to this paper.

The energy of formation of solitons in hexagonal clusters of aligned polyethylene molecules was estimated to be approximately 0.5 eV. Thermally assisted formation of solitons may explain the temperature and strain rate dependences of plasticity, creep and viscoelastic properties of the material. Dynamical simulations at finite temperatures will be very instructive, but kinetic Monte Carlo simulations may be preferable in view of the extremely high strain rates that pertain in molecular dynamics simulations.

### (c) Pull-out stresses

The assumption that the molecule is pulled out *en masse* as a rigid object was made to distinguish clearly this mechanism of plastic deformation from that involving solitons. It is analogous to the assumption of block slip in crystals which is made to estimate their theoretical shear strength [19]. In reality chain pull-out will occur at lower stresses than the theoretical values we have obtained here because solitons will be involved, just as dislocations are involved in slip in crystals. The finite formation energy of solitons will again lead to smaller pull-out stresses at higher temperatures and smaller strain rates.

### (d) Sensitivity analysis

We have carried out an analysis of the sensitivity of our results to the parameters assumed in table 1. Probably the most sensitive and uncertain is the length-scale *σ* in the LJ potential. Consider the effect of a 5% reduction of *σ* from 3.80 to 3.61 Å. This has no influence on the stiffness *κ* of the chain and the bead spacing *a* along the chain. But it changes the amplitude *V* of the sinusoidal potential in equation (3.4) from 1.035 to 1.899 meV. The equilibrium separation *ρ* of chains decreases from 4.124 to 3.838 Å, which in turn increases Young's modulus from 278 to 321 GPa. The theoretical pull-out stress for a chain of 10 μm length at the centre of a hexagon of aligned chains increases from 545 GPa to 6.91 TPa. The width of a soliton in such a hexagonal array decreases from 54 to 40 Å, and its energy of formation increases from 0.529 to 0.719 eV. The yield stress, as defined by equation (4.8), increases from 310 to 415 MPa. It is clear from this analysis that the absolute values of some of the calculated quantities are very sensitive to what is assumed for *σ*.

### (e) Outlook and validation

The straight chain model we have used here for polyethylene has the great advantage that it is amenable to analytic study. The more realistic zig-zag chain model introduces the torsion of the molecule as a further degree of freedom, which will certainly play an important role in determining both the ground state and the elementary excitations in aligned polyethylene. But the complexity of this model is such that it can be investigated only numerically through simulations. Nevertheless, it will provide improved estimates of the soliton properties we have discussed here.

One way to test the ideas in this paper is to carry simulations using the zig-zag chain model of aligned polyethylene molecules with finite lengths subjected to tensile loads. The chain ends should act as nucleation sites for solitons and twistons. The collective behaviour of these defects should account for the experimentally observed plasticity and viscoelasticity of aligned polyethylene, including hystereses in the stress–strain relation and the Bauschinger effect, and the strain rate and temperature dependences of the strength of the material.

## Supplementary Material

It is included in the main document

## Footnotes

1 The energy parameter *ϵ* is twice the value given in [11] for a CH_2_ group, as one bead represents two CH_2_ groups.

## Data accessibility

The data in figure 2 were published under a creative commons licence by Del Rosso *et al.* [20].
